# Noncovalent dyads of lanthanide nitride cluster fullerenes Ln_3_N@C_80_ and bisphthalocyanines LnPc_2_: Insights from DFT calculations

**DOI:** 10.1007/s00894-025-06415-7

**Published:** 2025-07-24

**Authors:** Lina M. Bolivar-Pineda, Elena V. Basiuk, Vladimir A. Basiuk

**Affiliations:** 1 Instituto de Ciencias Aplicadas y Tecnología, Universidad Nacional Autónoma de México, Circuito Exterior C.U., Ciudad de México, 04510 México; 2 Instituto de Ciencias Nucleares, Universidad Nacional Autónoma de México, Circuito Exterior C.U., Ciudad de México, 04510 México

**Keywords:** Lanthanides, Endohedral fullerenes, Bisphthalocyanines, Density functional theory, Geometries, Electronic parameters

## Abstract

**Context:**

Lanthanide-based systems, such as nitride cluster fullerenes Ln_3_N@C_80_ and bipthalocyanines LnPc_2_ (Pc = phthalocyanine ligand), are of interest for their magnetic, fluorescent and electronic properties. In this regard, we performed DFT characterization to investigate the changes in structure and electronic properties for noncovalently interacting lanthanide (Ln; where Ln = La, Ce, Gd and Lu) nitride cluster fullerenes and bisphthalocyanines to form Ln_3_N@C_80_ + LnPc_2_ dyads. The optimized geometries, formation and frontier orbital energies, HOMO-LUMO plots, charge and spin of Ln and N(NCF) atoms, as well as spin density plots of the dyads were analyzed in comparison with those of isolated Ln_3_N@C_80_ and LnPc_2_ components. In addition to LnPc_2_ bending distortion, the noncovalent dyad formation alters the geometry of the encapsulated Ln_3_N cluster, favoring more planar or pyramidal geometries, depending on the case. The HOMO and LUMO orbitals are found on bisphthalocyanines, being localized on the isoindole units, except for Ce_3_N@C_80_ + CePc_2_ dyad, where the LUMO was found on the central metal of CePc_2_. The HOMO-LUMO gap energy is lower for the dyads compared to isolated NCFs, being rather close to the gap energy of bisphthalocyanines. The changes in spin density distribution are evident in the dyads containing Ce and Gd atoms, contrary to their La and Lu-derived counterparts. The interaction of Ce_3_N@C_80_ and Gd_3_N@C_80_ with CePc_2_ and GdPc_2_, respectively, causes redistribution of the spin density, with changes in the orientation of spin-up and spin-down electrons in the encapsulated Ce_3_N and Gd_3_N clusters.

**Methods:**

The geometry optimization and electronic properties calculations based on density functional theory were performed using the DMol^3^ module of Material Studio 8.0 software package from Accelrys Inc. The computational parameters selected included the general gradient approximation functional PBE, combined with a long-range dispersion correction developed by Grimme (PBE-D2), the double numerical basis set (DN), equivalent to the 6-31G Pople-type basis set along with the DFT semiconductor pseudopotentials. To mitigate the self-consistent field convergence problems, the thermal smearing technique was applied, with a final very small value of 0.0001 Ha (equivalent to 31.6 K temperature), or Fermi orbital occupancy in some cases.

**Graphical Abstract:**

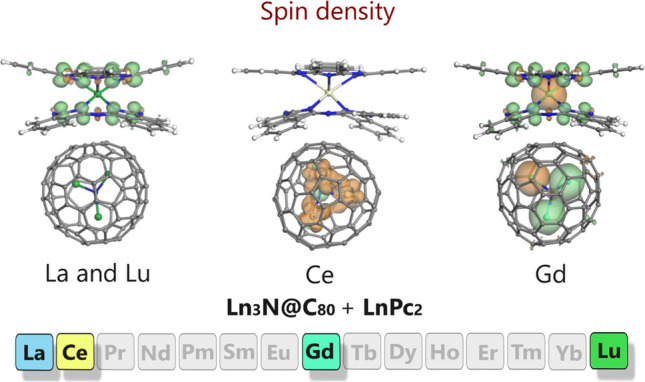

**Supplementary Information:**

The online version contains supplementary material available at 10.1007/s00894-025-06415-7.

## Introduction

Diverse systems including lanthanides (Ln; from La to Lu) are attracting increasing attention, particularly in the research and development of magnetic and luminescent materials [1, 2]. The unique electronic, optical and magnetic properties of lanthanide compounds have enabled their integration into a wide range of industries encompassing nuclear energy, electronics, machine-building, defense, chemical processing, and metallurgy. Some bright examples, where lanthanides play a crucial role, are such cutting-edge bio-imaging techniques as luminescence imaging, magnetic resonance imaging and tomography, as well as the development of optical, thermal, and gas sensors [3–5]. Among versatile lanthanide-based materials, lanthanide nitride cluster fullerenes (NCFs) and bisphthalocyanines (LnPc_2_) have emerged as particularly promising candidates for the related advanced applications.

**Fig. 1:**
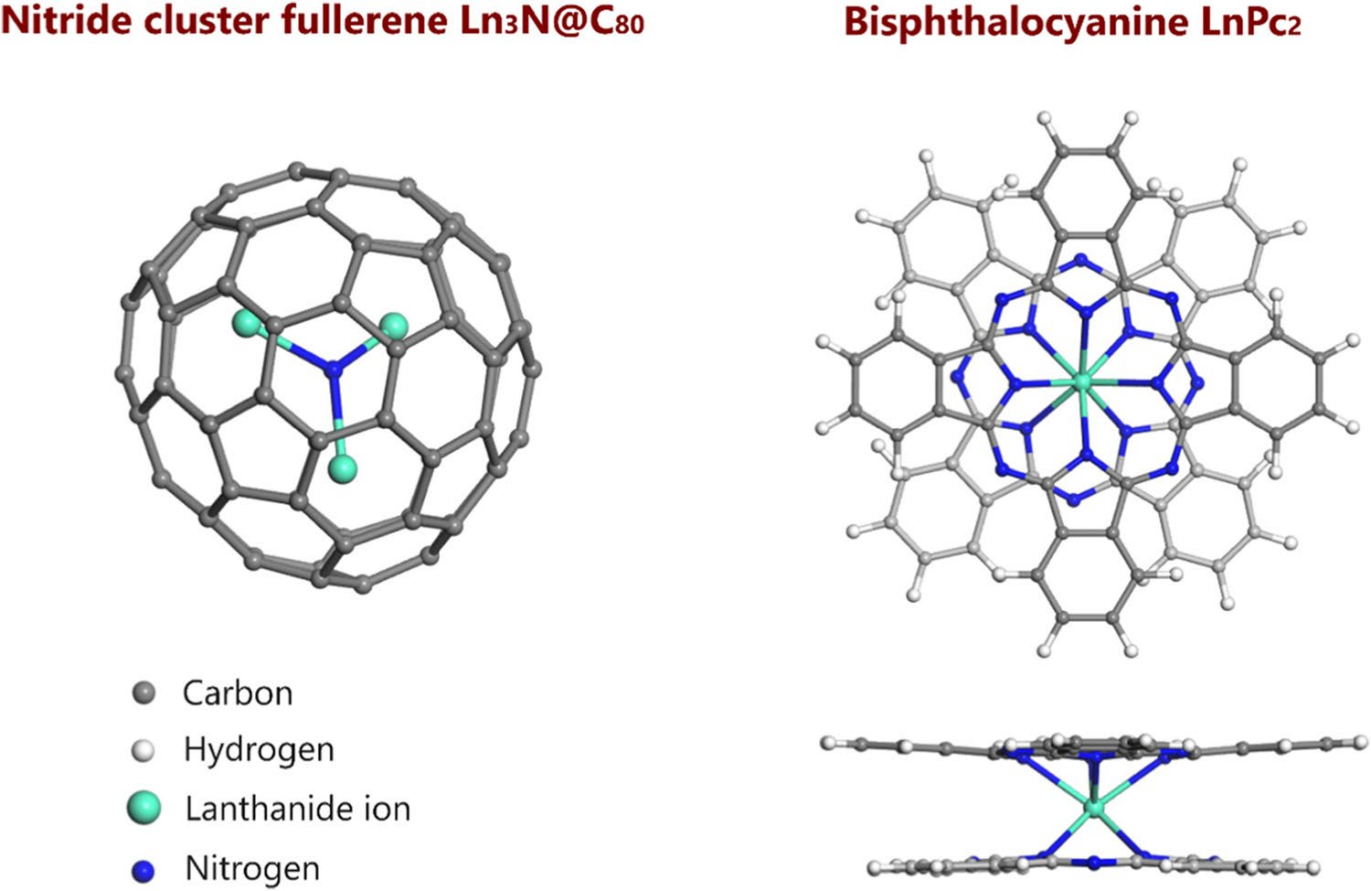
Optimized geometries of a lanthanide nitride cluster fullerene (left) and a lanthanide double-decker phthalocyanine (right, front and side views). Gadolinium NCF and Gd double-decker phthalocyanine (bisphthalocyanine) are shown as representative examples

NCFs are a type of endohedral fullerenes (EFs) in which a metal nitride cluster (M_3_N, where M is a rare-earth metal such as Sc, Y, or Ln) is encapsulated within a closed carbon cage, specifically a fullerene (C_*n*_, where *n* denotes the number of carbon atoms in the cage) [6, 7]. In the M_3_N cluster, M^3+^ ions are located at the vertices of a triangle with the nitride ion N^3-^ in its center (Fig. 1) [6]. NCFs can include homogeneous clusters (M_3_N@C_*n*_) or mixed-metal clusters (A_*x*_B_3-*x*_N@C_2*n*_, where x = 0–3, and A and B represent rare-earth ions), with a wide range of carbon cage sizes varying from C_68_ to C_88_ [7]. Among these, the M_3_N@C_80_ (where C_80_ has *I*_h_ symmetry) compounds is the most extensively studied and widely explored family of NCFs. In this group, the metal nitride cluster and the C_80_ fullerene cage stabilize each other through the transfer of six electrons from the nitride cluster to the fullerene cage [7]. One of the most captivating features of most endohedral fullerenes containing lanthanide ions is their incomplete 4*f* electron shell (except for Lu derivatives). This configuration leads to the formation of substantial magnetic moments and a range of intriguing magnetic features, which significantly increase the potential for various applications of these EFs [7, 8].

On the other hand, the lanthanide bisphthalocyanines are conjugated macrocycles consisting of a (usually - except for Ce^4+^) trivalent central metal Ln^3+^, a dianionic macrocycle (Pc^2-^, where Pc stands for phthalocyanine ligand), and a monoanionic radical ligand (Pc•-), forming the structure [Ln^3^⁺(Pc^2^⁻)(Pc•⁻)] [9]. These complexes are characterized by their unique"sandwich-type"structure, where the central metal ion is coordinated to eight N atoms belonging to the two phthalocyanine ligands (Fig. 1). The bisphthalocyanines exhibit distinctive electronic and magnetic properties, making them especially promising as single-molecule magnets (SMMs) [10, 11].

The formation of hybrids or dyads of carbon nanomaterials, such as graphene and carbon nanotubes, with lanthanide bisphthalocyanines were demonstrated to enhance the magnetic properties due to synergy between the two components [10, 12–15]. These improvements are particularly attractive for future applications in spintronic devices [10, 12–15]. One should also mention that the π–extended compounds (especially polyazamacrocycles), exemplified by porphyrins (Ni(II) or Co(II) octaethylporphyrins (OEPs) [16, 17] Zn(II), tetraphenyl-porphyrin [18]), decapyrrylcorannulene (DPC) [17, 19], Ni (II) tetramethyldibenzo-tetraazaannulenato [20] and octamethyldibenzo-tetraazaannulenato [21] serve as the first-choice crystallizing agents for endohedral fullerenes, enabling their structural characterization by means of X-ray diffraction, since most EFs exhibit serious challenges in crystallizing in their neat form. The complexation of this sort can alter the EFs structure; however, it is unclear to what extent. Some insight can be afforded from density functional theory (DFT) calculations, as exemplified by the recent analysis of the changes in structure and electronic properties of Sc_3_N@C_80_ NCF induced by its interaction with the above-mentioned crystallizing agents [22].

From various aspects mentioned above, we found it interesting and important to undertake the present DFT characterization of the changes in structure and electronic properties for noncovalently interacting Ln_3_N@C_80_ and LnPc_2_ molecules to form Ln_3_N@C_80_ + LnPc_2_ dyads. As representatives of the lanthanide series, we selected lanthanum (La), cerium (Ce), gadolinium (Gd) and lutetium (Lu). These lanthanides were chosen due to their variable 4*f* electronic configurations. Lanthanum ion La^3+^, with no 4*f* electrons (4*f*^0^), serves as a reference point for the absence of 4*f* contributions; gadolinium, Gd^3+^, has a half-filled 4*f* electrons (4*f*^7^); and lutetium, Lu^3+^, has a fully filled *4f* orbital (4*f*^14^). Also, we included a very peculiar case of cerium, whose electronic configuration is different in Ce_3_N@C_80_ and CePc_2_: in the former, its ions Ce^3+^ have one 4*f* electron (4*f*^1^), whereas in the latter, the ion Ce^4+^ has no 4*f* electrons, thus making the corresponding bisphthalocyanine the only closed-shell exception from the LnPc_2_ series of SMMs.

## Computational methods

The geometry optimization and calculations of selected electronic properties for the Ln_3_N@C_80_ + LnPc_2_ dyads was carried out using the DMol^3^ DFT module available within the Materials Studio 8.0 software package (Accelrys, Inc) [23, 24]. The computations were conducted within the frame of the generalized gradient approximation (GGA) with the Perdew-Burke-Ernzerhof (PBE) functional [25]. To better account for dispersion interactions, which are critical for systems involving noncovalent interactions such as fullerene and phthalocyanine dyads, a long-range dispersion correction (usually referred to as PBE-D2) was included [26]. This approach goes in line with existing studies on tetraazamacrocycles (porphyrins and Pcs) [27–31], graphene [32, 33], endohedral fullerenes [22, 25, 34–37] and carbon nanotubes [38–41].

Based on the recent studies performed by our group [42, 43], which explored the characteristic of lanthanide bisphthalocyanines LnPc_2_ and lanthanide nitride cluster fullerenes Ln_3_N@C_80_, treated independently, we followed the same methodology for analyzing their Ln_3_N@C_80_ + LnPc_2_ dyads. To address the challenges posed by the presence of *4f*-electrons in terms of the serious difficulties to achieve self-consistent field (SCF) convergence, the calculations employed pseudopotentials instead of full-electron treatment. Of the two types incorporated in the DMol^3^ module, which are the DFT semi-core pseudopotentials (DSPP) and effective core potentials (ECP), we chose DSPP since, in addition to incorporating relativistic effects and spin-orbit coupling, they were specially developed to use within the present module, as well as allow to treat successfully all lanthanides including the ‘difficult’ terbium [44]. Furthermore, in an even more recent study of the Ln interactions with some defect-containing graphene models [45] we found that the calculations can produce a negatively (unlike all other lanthanides) charged Dy atom from the Mulliken population analysis, or at best a very small and unrealistic positive charge from the Hirshfeld analysis.

The computational parameters included a double numerical basis set (DN), equivalent to 6-31G Pople-type basis set, along with a global orbital cutoff of 4.3 Å [46]. The lanthanide species considered have the following electronic configuration: La^3+^, [Kr] 5*s*^2^4*d*^10^5*p*^6^; Ce^3+^, [Xe] 4*f*^1^; Gd^3+^, [Xe] 4*f*^7^; Lu^3+^, [Xe] 4*f*^14^. The geometry optimization and electronic parameter calculations were performed with the convergence criteria defined as follows: the energy gradient of 10^−4^ Ha/Å, the maximum forces of 0.02 Ha/Å, the maximum displacement of 0.05 Å, and SCF tolerance 10^−4^. As is typical for the lanthanide-containing systems, the presence of a large number of degenerate states dramatically complicates (or sometimes even makes impossible) reaching SCF convergence; therefore, the thermal smearing technique was applied to overcome the SCF convergence issues, as it was previously explained in detail in Refs. [42, 43] and [47], that is, by means of the step-by-step reducing the smearing value starting with the default value of 0.005 Ha (equivalent to 1578.9 K temperature). The final results reported herein correspond to the smearing value of 0.0001 Ha (equivalent to 31.6 K temperature) in the case of Ln_3_N@C_80_ + LnPc_2_ dyads; furthermore, we were able to apply Fermi occupancy (no smearing) for all isolated LnPc_2_ species, La_3_N@C_80_, Gd_3_N@C_80_, and Lu_3_N@C_80_. (Among other tools which can help to mitigate the problems with SCF convergence are the direct inversion of the iterative subspace, or DIIS, the charge density preconditioner, as well as the charge and spin density mixing; the corresponding values we set in the calculations were 10, 10, 0.1 (0.2 for Lu) and 0.1 (0.2 for Lu), respectively.) The maximum number of both SCF iterations and optimization cycles was adjusted (by manually editing the input files) to 10,000.

Formation energies ${\Delta E}_{i}$ for Ln_3_N@C_80_ + LnPc_2_ dyads models were calculated using the equation:

$${\Delta E}_{{\text{Ln}}_{3}\text{N}@{\text{C}}_{80}+{\text{LnPc}}_{2} }= {E}_{{{\text{Ln}}_{3}\text{N}@{\text{C}}_{80}+{\text{LnPc}}_{2}}}-\left({E}_{{\text{Ln}}_{3}\text{N}@{\text{C}}_{80}}+ {E}_{{\text{LnPc}}_{2}}\right)$$

where ${E}_{i}$ represents the absolute energy of the respective system. The values for ${E}_{{\text{Ln}}_{3}\text{N}@{\text{C}}_{80}}$ and ${E}_{{\text{LnPc}}_{2}}$ were reported in our previous studies [42, 43]. The corresponding values, as well as the most important geometric and electronic parameters for the isolated bisphthalocyanines and the endohedral fullerene component are summarized in the Supplementary information file.

## Results and discussion

**Fig. 2:**
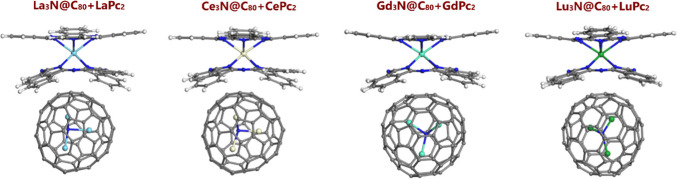
Optimized geometries of Ln_3_N@C_80_ + LnPc_2_ (Ln = La, Ce, Gd and Lu) dyads. The atoms are colored as follows: carbon in gray, hydrogen in white, nitrogen in deep blue, lanthanum in light blue, cerium in light yellowish-white, gadolinium in turquoise blue, and lutetium in green

Four molecular models of Ln_3_N@C_80_ + LnPc_2_ dyads were built, where Ln represents lanthanum, cerium, gadolinium, and lutetium. Each one of the dyads contains four lanthanide ions. In these systems, the C_80_ fullerene hosts a homogeneous Ln_3_N cluster, meaning the lanthanide atoms within the cluster are identical. Furthermore, in three dyads, the lanthanide ion of the bisphthalocyanines is the same and in the same oxidation state as those which compose the endohedral cluster within the fullerene cage C_80_ (Fig. 2): this is the case for La^3+^, Gd^3^+ and Lu^3+^. The exception is Ce_3_N@C_80_ + CePc_2_ dyad, in which Ce_3_N@C_80_ contains Ce^3+^ ions, whereas CePc_2_, Ce^4+^ ion.

The structural characteristics, energy formation, and electronic properties of Ln_3_N@C_80_ + LnPc_2_ dyads were analyzed by comparing them with the features of their isolated components, namely LnPc_2_ and Ln_3_N@C_80_, which were previously reported [42, 43]. (We would like to briefly mention an unsuccessful attempt of the calculations of Tb_3_N@C_80_ + TbPc_2_ dyad. In this particular case, the SCF convergence was impossible to achieve even at the highest thermal smearing value of 0.005 Ha (equivalent to 1578.9 K temperature), regardless of the values of DIIS, charge density preconditioner, charge and spin density mixing used.)

Fig. 2 illustrates the optimized geometry of Ln_3_N@C_80_ + LnPc_2_ dyads, revealing that the bisphthalocyanine shape undergoes distortion additional to its intrinsic characteristic geometry (Pc ligands with a domed or non-planar structure [40]) when interacting with the lanthanide nitride cluster fullerene. This distortion arises from the tendency to maximize contact area between the phthalocyanines and carbon nanomaterials with curved surfaces, such as carbon nanotubes [39, 40], graphene with structural defects [48], and in the present case the spherical surface of Ln_3_N@C_80_. For the common free-base phthalocyanine H_2_Pc and its 3*d*-transition metal complexes, which are planar in the isolated state, it was systematically observed previously that the fullerene cage occupies a centered position with respect to the N_4_ coordination sphere (directly interacting with the metal, if it is present) [25, 35–37, 49, 50]. But in the present case, the concave surface of LnPc_2_ is even a better match of the spherical Ln_3_N@C_80_ molecule. As a result, even though the two interacting molecules were not perfectly aligned in the starting geometry, it did not affect the final optimized geometries presented in Fig. 2.

Structural parameters of the four dyads were analyzed to identify whether there was any alteration of the Ln_3_N@C_80_ geometry due to the interaction with the bisphthalocyanines. For this purpose, the structure of Ln_3_N@C_80_ in the dyads was compared with the structure of isolated Ln_3_N@C_80_. Table 1 and Table S1 (Supplementary Information) presents the values of the Ln-N bond lengths, the Ln-N-Ln angles, and the pyramidalization angles (θ) of the Ln_3_N cluster, as well as the closest distance between LnPc_2_ and Ln_3_N@C_80_ for each dyad, as additional important characteristics.

**Table 1:** Ln-N bond lengths (in Å), Ln-N-Ln angles (in °), the pyramidalization angle (θ) of the Ln_3_N cluster (in °) encapsulated in C_80_ fullerene, noncovalently interacting with lanthanide bisphthalocyanines. Also included are the shortest distances (in Å) between the components of Ln_3_N@C_80_ + LnPc_2_ dyads, such as ${N}_{{LnPc}_{2}}$···${C}_{{C}_{80}}$, ${C}_{{LnPc}_{2}}$···${C}_{{C}_{80}}$ and ${Ln}_{{LnPc}_{2}}$···${C}_{{C}_{80}}$

Dyads	Ln_3_N@C_80_			Closest contacts (Å)		
	Ln-N (Å)	Ln-N-Ln (°)	θ (°)	${{N}}_{{{L}{n}{P}{c}}_{2}}$··· ${{C}}_{{{C}}_{80}}$	${{C}}_{{{L}{n}{P}{c}}_{2}}$··· ${{C}}_{{{C}}_{80}}$	${{L}{n}}_{{{L}{n}{P}{c}}_{2}}$··· ${{C}}_{{{C}}_{80}}$
La_3_N@C_80_ + LaPc_2_	2.237, 2.257, 2.265	96.4, 97.5, 98.6	29.7	2.975	2.889	4.271
Ce_3_N@C_80_ + CePc_2_	2.147, 2.136, 2.165	103.7, 104.1, 106.5	23.8	2.979	2.904	4.190
Gd_3_N@C_80_ + GdPc_2_	2.094, 2.110, 2.110	110.3, 114.0, 114.1	15.9	2.884	2.967	4.250
Lu_3_N@C_80_ + LuPc_2_	2.048, 2.062, 2.063	115.5, 121.4, 121.6	4.1	2.948	2.946	4.055

**Fig. 3:**
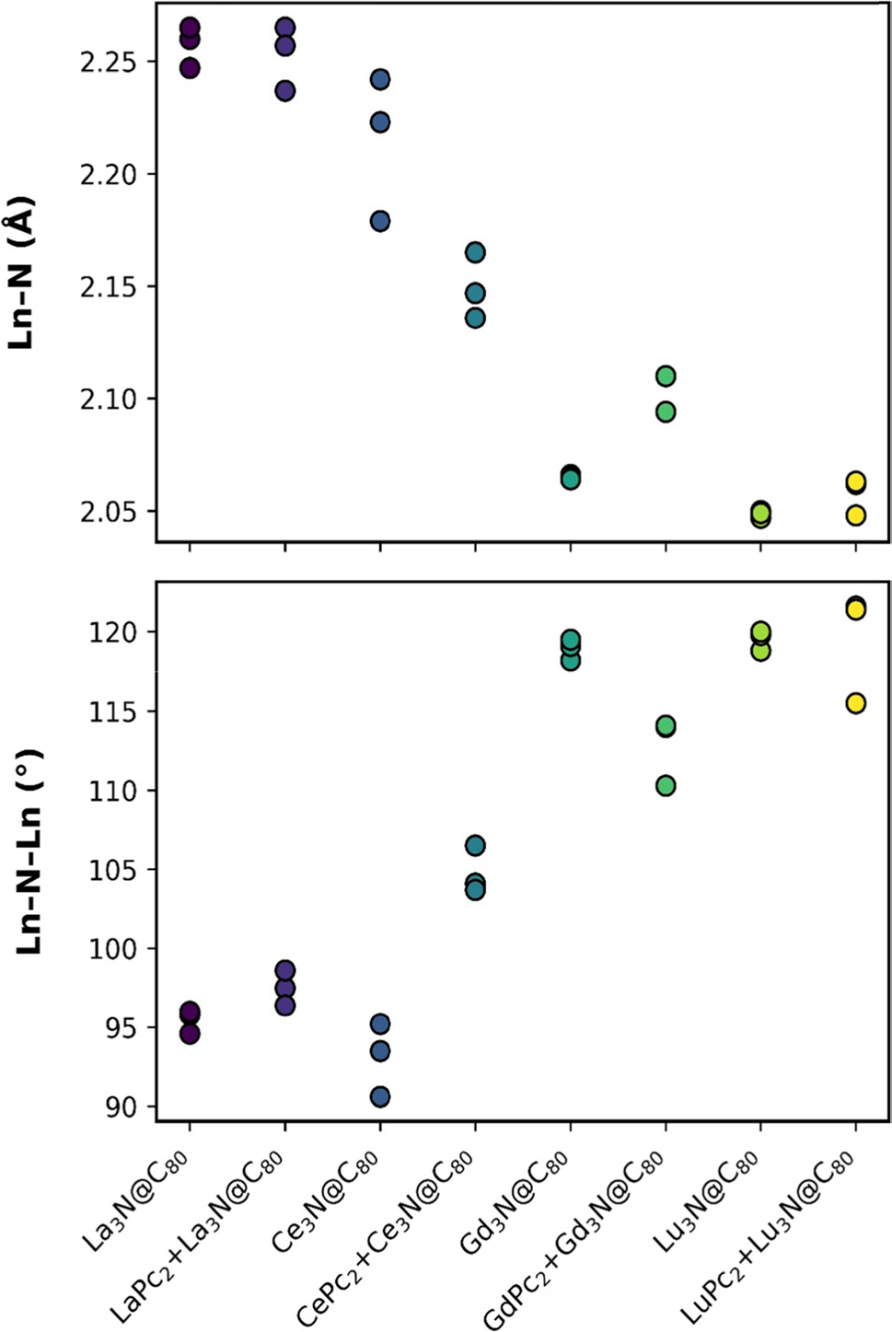
Comparison of the Ln-N bond lengths (Å; top) and Ln-N-Ln angles (°; bottom) in the isolated lanthanide nitride cluster Ln_3_N@C_80_ (Ln = La, Ce, Gd, and Lu; the values reported in Ref. [42]) and in the noncovalent Ln_3_N@C_80_ + LnPc_2_ dyads

The trends in changes in the Ln-N bond lengths and the Ln-N-Ln angles of the Ln_3_N cluster encapsulated in the C_80_ fullerene of the dyads is illustrated by Fig. 3. For La_3_N@C_80_ + LaPc_2_, the Ln-N bond length range was 2.237 to 2.265 Å. Although some differences with the isolated fullerene cluster were detected, such as the contraction of one of the La-N bonds, it can be inferred that the interaction with LaPc_2_ does not significantly alter the cluster bonds within the fullerene. Meanwhile, the Ln-N bond length range for Ce_3_N@C_80_ + CePc_2_ was 2.136 to 2.165 Å, for Gd_3_N@C_80_ + GdPc_2_ was 2.094 to 2.110 Å, and for Lu_3_N@C_80_ + LuPc_2_ was 2.048 to 2.063 Å. Particularly for these dyads with Ce, Gd and Lu, significant changes were noted. In Ce_3_N@C_80_ + CePc_2_, the Ce-N bond length decreased, while in Gd_3_N@C_80_ + GdPc_2_ and Lu_3_N@C_80_ + LuPc_2_, the bond lengths increased, with Gd_3_N@C_80_ + GdPc_2_ showing the most noticeable increase. In the latter two cases, the equidistant nature of the bond lengths in the Gd_3_N and Lu_3_N clusters was lost, compared to their respective isolated NCF structures.

The Ln-N-Ln angles and the pyramidalization angle are fundamental parameters to characterize the geometry of the Ln_3_N cluster within the fullerene. The pyramidalization angle measures the deviation of the central nitrogen atom from an ideal planar configuration, so that an angle greater than 0° indicates greater distortion of planarity, resulting in a pyramidal structure, whereas an angle equal to 0° is indicative of a completely planar arrangement [6, 51]. Also, it is relevant to consider that the size of the lanthanide metal ionic radius also influences this feature [6, 51]. Clusters containing smaller size metals, such as lutetium (0.861 Å), tend to present a planar or relatively planar geometry, while larger ones, such as lanthanum (1.06 Å), adopt a pyramidal geometry. In the hybrids, both parameters displayed variations when phthalocyanines were present (Fig. 3 and Table S1).

For La_3_N@C_80_ + LaPc_2_, Ln-N-Ln angles ranged between 96.4° and 98.6°, and between 103.7° and 106.5° for Ce_3_N@C_80_ + CePc_2_, showing a more significant increase for the latter, along with an alteration in its θ (Fig. 3, Table 1 and Table S1). Compared to the isolated NCFs, the dyads with La and Ce showed the reduction by 1.6° and 9.2°, respectively, but both clusters maintained their distorted pyramidal geometry, with Ln-N-Ln angles less than 120°. Regarding Gd_3_N@C_80_ + GdPc_2_, the Ln-N-Ln angles ranged from 110.3° to 114.1°, showing a decrease, while for Lu_3_N@C_80_ + LuPc_2_, the values were from 115.5° to 121.6°. As can be seen in Fig. 3, no clear trend is apparent, since two angles increased and one decreased. As for the θ, it increased by 10° for the dyad with Gd and by 0.2° for the dyad with Lu. This suggests that, in the Gd_3_N@C_80_ + GdPc_2_ dyad, the geometry of Gd_3_N experienced a loss of planarity, while Lu_3_N suffered minimal distortion in its trigonal planar geometry.

The interaction between lanthanide bisphthalocyanines and lanthanide nitride cluster fullerenes was numerically characterized by the closest intermolecular contacts ${\text{N}}_{{\text{LnPc}}_{2}}$···${\text{C}}_{{\text{C}}_{80}}$, ${\text{C}}_{{\text{LnPc}}_{2}}$···${\text{C}}_{{\text{C}}_{80}}$ and ${\text{Ln}}_{{\text{LnPc}}_{2}}$···${\text{C}}_{{\text{C}}_{80}}$ (Table 1). The shortest distances for La_3_N@C_80_ + LaPc_2_ (2.889 Å), Ce_3_N@C_80_ + CePc_2_ (2.904 Å), and Lu_3_N@C_80_ + LuPc_2_ (2.946 Å) corresponded to interactions between carbon atoms of both components (${\text{C}}_{{\text{LnPc}}_{2}}$···${\text{C}}_{{\text{C}}_{80}})$. In the case of Gd_3_N@C_80_ + GdPc_2_ (2.884 Å), the closest contact occurred between the nitrogen atom of the isoindole unit (that is, the nitrogen coordinated to the lanthanide metal) and a carbon atom of the fullerene C_80_. It is worth noting that the interaction of the nitrogen from the isoindole unit also occurs in the other three remaining dyads; however, it represents the second shortest distance, following the ${\text{C}}_{{\text{LnPc}}_{2}}$···${\text{C}}_{{\text{C}}_{80}}$ interaction. The shortest distance between the lanthanide metal of the bisphthalocyanine and a carbon atom of the cage ranges from 4.055 to 4.271 Å, with the largest distance observed for La_3_N@C_80_ + LaPc_2_ and the smallest for Lu_3_N@C_80_ + LuPc_2_.

The formation energy of the Ln_3_N@C_80_ + LnPc_2_ dyads (Table 2) is the most direct measure of the interaction strength between the components, influenced by the electronic configurations and π-π interactions. The values obtained evidence that the most stable system is Gd_3_N@C_80_ + GdPc_2_ with a formation energy of −45.66 kcal/mol, followed by Lu_3_N@C_80_ + LuPc_2_ with −44.43 kcal/mol, La_3_N@C_80_ + LaPc_2_ with −44.06 kcal/mol and finally Ce_3_N@C_80_ + CePc_2_ with −38.61 kcal/mol. In other words, the system with gadolinium, characterized by its 4*f* half-full electron configuration, has the most negative formation energy value, reflecting the strongest interaction. In contrast, the system with cerium, which possesses a single 4*f* electron in Ce_3_N@C_80_ and no 4*f* electrons at all in CePc_2_, exhibits the lowest relative stability. Table S2 presents the data on the total energy and formation energy for each isolated component, as well as for the dyads.

**Table 2:** Formation energies (Δ*E* in kcal/mol), HOMO, LUMO and HOMO-LUMO gap energies (in eV) for Ln_3_N@C_80_ + LnPc_2_ dyads, as well as the charge and spin (in *e*) for the Ln and N atoms in Ln_3_N cluster and for the Ln atom in LnPc_2_ molecule

Dyads	Δ*E* (kcal/mol)	HOMO (eV)	LUMO (eV)	*E*_gap _(eV)	Ln_3_N@C_80_				LnPc_2_	
					Ln charge (***e***)	N charge (***e***)	Ln spin (***e***)	N spin (***e*)**	Ln charge (***e***)	Ln spin (***e***)
La_3_N@C_80_ + LaPc_2_	−44.06	−5.011	−4.880	0.131	0.512, 0.721, 0.484	−0.946	0, 0, 0	0	1.903	0
Ce_3_N@C_80_ + CePc_2_	−38.61	−4.850	−4.661	0.189	0.638, 0.421, 0.473	−0.897	−1.019, −1.019, −1.036	0.093	1.779	−0.001
Gd_3_N@C_80_ + GdPc_2_	−45.66	−4.922	−4.791	0.131	0.557, 0.568, 0.502	−0.955	−6.906, 6.944, 6.988	−0.028	1.474	−7.011
Lu_3_N@C_80_ + LuPc_2_	−44.43	−4.869	−4.735	0.135	0.636, 0.602, 0.688	−1.046	0, 0, 0	0	1.428	0.002

The electronic properties of the Ln_3_N@C_80_ + LnPc_2_ dyads were evaluated in terms of the energies and distribution of frontier orbitals HOMO and LUMO, HOMO-LUMO gap energies (*E*_gap_), the spin density distribution within the dyad, the charge and spin state of the lanthanide and nitrogen, obtained from the Mulliken population analysis. (We prefer the latter type of population analysis since it was shown to provide an adequate characterization of the charge on the lanthanide atom [52–54]. Additionally, it was demonstrated that the use of a smaller basis set, like the DN employed in the present study, produces more reliable results for Mulliken population analysis [55–57].)

**Fig. 4:**
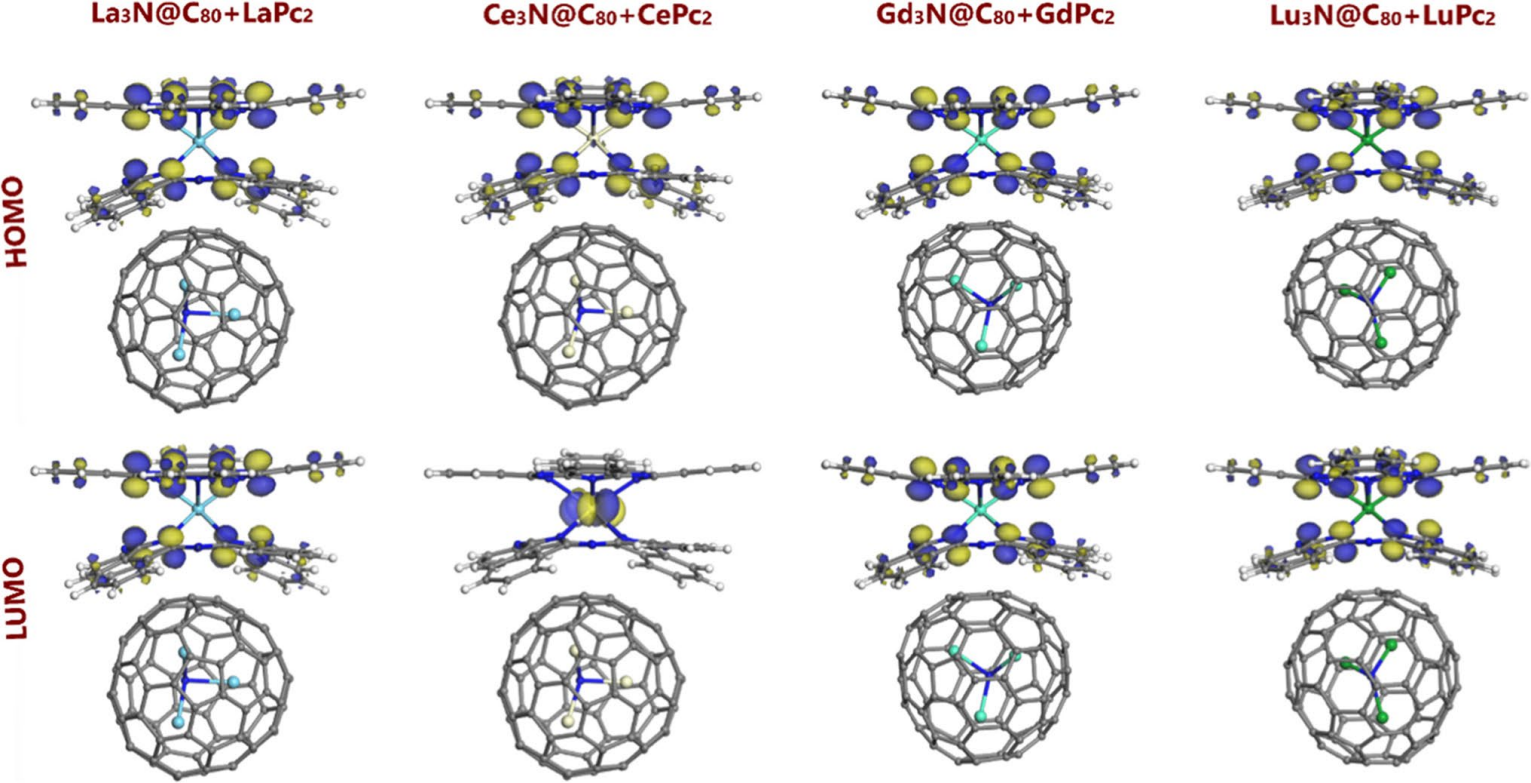
Frontier orbital distribution (HOMO and LUMO; isosurfaces at 0.03 a.u.) for noncovalent Ln_3_N@C_80_ + LnPc_2_ dyads

The HOMO and LUMO isosurfaces are shown in the Fig. 4. In most dyads, the largest lobes of both HOMO and LUMO are localized on the carbon atoms of isoindole units of Pc ligands. At the same time, in the dyad with cerium, the LUMO lobes are found on the central metal: this can be attributed to the highest oxidation state 4+ characteristic for Ce in CePc_2_. In the isolated NCFs La_3_N@C_80_ and Ce_3_N@C_80_, the LUMO lobes are clearly localized on all four Ln_3_N atoms, contrary to the cases of Gd and Lu [42]. As follows from Table 2, the HOMO-LUMO gap of the dyads is smallest for the La and Gd derivatives, of 0.131 eV. These are followed by Lu_3_N@C_80_ + LuPc_2_ with an *E*_gap_ of 0.135 eV, and Ce_3_N@C_80_ + CePc_2_, of 0.189 eV. These values closely match the ones calculated previously for the respective isolated bisphthalocyanines [43]; in other words, it is LnPc_2_ component, which is responsible for the reactivity of the entire dyad.

**Fig. 5:**
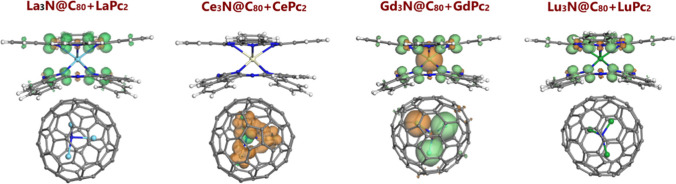
Spin density patterns for noncovalent Ln_3_N@C_80_ + LnPc_2_ dyads (isosurfaces at 0.01 a.u.). The green and orange lobes correspond to spin-up and spin-down electrons, respectively

The analysis of spin density distribution is of special importance for lanthanide-containing systems, as it provides essential details about the distribution and delocalization of unpaired electrons. This aspect is crucial for understanding magnetic behavior, especially in the molecules which exhibit SMM properties, such as bisphthalocyanines, where the magnetic response is strongly influenced by the distribution of electrons within the isolated molecules and/or hybrids/dyads [10, 11]. In the present particular case of four Ln_3_N@C_80_ + LnPc_2_ dyads considered, we found actually three different patterns (Fig. 5). For La_3_N@C_80_ + LaPc_2_ and Lu_3_N@C_80_ + LuPc_2_, the spin density plots are generally identical, where unpaired electrons (among which the spin-up orientation strongly dominates) are located exclusively (at least in the isosurfaces at 0.01 a.u.) on the Pc ligands. This picture closely matches the corresponding HOMO and LUMO isosurfaces. The dominating spin-up orientation (the green lobes in Fig. 5) is mainly associated with the carbon atoms of the isoindole units of LnPc_2_, whereas the spin-down electron density (the orange lobes in Fig. 5) is found on the nitrogen atoms of the macrocyclic ligands, making a minor contribution. In this way, the spin density distribution remains essentially identical with that observed in the isolated LaPc_2_ and LuPc_2_ molecules [43]. In contrast, the isolated La_3_N@C_80_ and Lu_3_N@C_80_ behave (and indeed are) as closed-shell systems, and this feature remains obvious for the La_3_N@C_80_ + LaPc_2_ and Lu_3_N@C_80_ + LuPc_2_ dyads (the spin density plots of the isolated bisphthalocyanines, NCFs and their dyads are compared in Fig. S1).

In the Ce and Gd-derived dyads, new important and interesting features can be revealed, which are naturally linked to the presence and number of 4*f* electrons. To begin with, even though the formation of noncovalent dyads Ce_3_N@C_80_ + CePc_2_ and Gd_3_N@C_80_ + GdPc_2_ does not give rise to changes in the localization of unpaired electrons, notable changes can be seen in their orientation, compared to the plots characteristic for the individual components. As it was already mentioned above, the case of cerium is very peculiar, since its oxidation state in Ce_3_N@C_80_ and CePc_2_ is not the same. The NCF contains three Ce^3+^ ions, each of them having one 4*f* electron. The bisphthalocyanine complex contains one Ce^4+^ ion (with no 4*f* electrons), which fully compensates the charge of four protons of two H_2_Pc molecules: thus, CePc_2_ becomes a closed-shell (not a radical) system, becoming a “black sheep” in the rare-earth SMM family. This explains the absence of even minor lobes in the spin density plots of both isolated CePc_2_ and same bisphthalocyanine in Ce_3_N@C_80_ + CePc_2_ dyad. As to Ce_3_N@C_80_, both the isolated and dyad-forming NCF exhibit dominating lobes on Ce atoms, which a much smaller contribution from the nitride N atom. But the spin orientation does not remain the same. In the dyad, the spin-up electrons are localized on the central nitrogen atom of the cluster, while the spin-down electrons extend to all three Ce atoms (Fig. 5). At the same time, in the isolated Ce_3_N@C_80_, the spin-up orientation can be seen on two Ce atoms, whereas the spin-down electrons are localized on the central N and one Ce atom (Fig. S1) [42].

Concerning the case of Gd_3_N@C_80_ + GdPc_2_, one can say that in a sense this dyad combines the features described for the other three dyads. The lobes of a broadly variable size can be found throughout structural elements of both components (Fig. 5). The spin density plots for GdPc_2_ both for the isolated molecule and for the dyad (which do not exhibit tangible differences) have much in common with the plots for the LaPc_2_ and LuPc_2_ analogues: namely, the distribution and orientation of unpaired electron density within the Pc ligands is almost identical. However, the central Gd atom having seven 4*f* electrons contributes with a new, much larger spin-down lobe. Each gadolinium atom in Gd_3_N@C_80_ unit makes a similar contribution, both in the isolated NCF and in the dyad, but in none of the two cases the spin direction coincides for all three atoms (contrary to the cerium atoms in Ce_3_N@C_80_ + CePc_2_). Namely, in isolated Gd_3_N@C_80_ (Fig. S1) [42], two of the three Gd atoms have spin-down orientation of unpaired electrons, whereas in the dyad the situation is opposite. The lobes on the central N atom are incomparably smaller compared to the ones found on Gd atoms, and their orientation changes as well: from spin-up in the isolated NCF to spin down in Gd_3_N@C_80_ + GdPc_2_ dyad. Even closer inspection of both Gd_3_N@C_80_ plots detects additional tiny lobes on some carbon atoms of the C_80_ cage, which manifested themselves in none of the other three cases, including the one of cerium.

One should mention that the behavior of GdPc_2_ was previously studied in the context of its noncovalent interactions with carbon nanotubes (with both zigzag and armchair chirality) and graphene (both defect-free and defect-containing), where the changes in spin distribution were analyzed in a similar way (in particular, switching between ferromagnetic and antiferromagnetic spin ordering was observed in some systems) [39, 48]. Unlike the present case of GdPc_2_ complexation with Gd_3_N@C_80_, in those ones, the changes in spin orientation occurred within the bisphthalocyanine component. This suggests that both the nature of Gd-containing component and the nature of carbon nanomaterial can influence the distribution and orientation of unpaired electrons, which in the end determines magnetic properties of the system.

The spin values characterize quantitatively the imbalance between the number of electrons with spin-up and spin-down orientations in a given atom/system. In line with the spin density plots, the atomic spin values for Ln_3_N units in the dyads with cerium and gadolinium showed detectable alterations in both magnitude and orientation, compared to their isolated counterparts (Table S3). Table 2 shows that the spin values for the dyads were negative (spin-down) for all three Ce ions in Ce_3_N and one Gd ion in Gd_3_N, whereas in the isolated Ce_3_N@C_80_ only one Ce ion exhibited spin-down electron orientation, and in the isolated Gd_3_N@C_80_, two Gd^3+^ ions. The spin direction on the N atom changed in both cases, but the absolute spin value, only in the case of Ce_3_N@C_80_ (from 0.042 *e* in the isolated NCF to 0.093 *e* in the dyad). For Gd_3_N@C_80_ and Gd_3_N@C_80_ + GdPc_2_, it remains the same of 0.028 *e*. As regards the spin values of Ln atoms in the bisphthalocyanines, the changes turned to be extremely insignificant, if any.

Finally, Table 2 and Table S3 specify the charge of Ln in each dyad and in their individual components, as well as the one of N atoms in each NCF. Overall, the results indicated that the interaction of LnPc_2_ with the corresponding Ln_3_N@C_80_ gives rise to an increase in the Ln charge in the bispthalocyanines, ranging from 0.028 *e* (for Lu_3_N@C_80_ + LuPc_2_) to 0.076 *e* (for La_3_N@C_80_ + LaPc_2_). Likewise, the charge of Ln atoms in NCFs increases due to the dyad formation, in some cases by more than 0.2 *e* (for example, 0.486 *e* for La_3_N@C_80_ *versus* 0.721 *e* in the corresponding dyad). As for the charge of the N atom, it becomes more negative in the La and Ce-derived dyads, by 0.021 *e* and 0.088 *e*, respectively. As opposite, in the systems with Gd and Lu, the negative charge on N is reduced by 0.037 *e* and 0.028 *e*, respectively.

## Conclusions

In addition to the bisphthalocyanine bending distortion, the noncovalent interaction of LnPc_2_ molecules with the related nitride cluster fullerenes Ln_3_N@C_80_ induces structural changes, which alter the geometry of the encapsulated Ln_3_N cluster, favoring more planar or pyramidal geometries, depending on the case. In the dyads with Gd and Lu, for example, a loss of planarity was observed. Furthermore, the electronic properties are modified as well. The HOMO and LUMO orbitals are found on the bisphthalocyanines, being localized on the isoindole units, with the exception of Ce_3_N@C_80_ + CePc_2_ dyad, where the LUMO was found on the central metal of CePc_2_. The HOMO-LUMO gap energy is lower for the dyads compared to isolated NCFs, being rather close to the gap energy of bisphthalocyanines. Thus, the electronic characteristics of Ln_3_N@C_80_ + LnPc_2_ dyads tend to be defined by the ones of the bisphthalocyanine component.

The changes in spin density distribution are evident in the dyads containing Ce and Gd atoms, contrary to their La and Lu-derived counterparts. The interaction of Ce_3_N@C_80_ and Gd_3_N@C_80_ with CePc_2_ and GdPc_2_, respectively, causes redistribution of the spin density, with changes in the orientation of spin-up and spin-down electrons in the encapsulated Ce_3_N and Gd_3_N clusters. Such a behavior might open the possibility to tune magnetic properties of these and similar dyads, in order to optimize their performance as SMMs in magnetic and/or spintronic devices. 

## Supplementary Information

Below is the link to the electronic supplementary material.

Supplementary Material 1 (DOCX 595 KB)

## Data Availability

Data will be made available on request. Data is provided within the manuscript and supplementary information files.
