# Supplementary material for: Noncovalent dyads of lanthanide nitride cluster fullerenes Ln3N@C80 and bisphthalocyanines LnPc2: Insights from DFT calculations
Source: J Mol Model. 2025 Jul 24;31(8):216. doi: 10.1007/s00894-025-06415-7 (PMC12287152; doi:10.1007/s00894-025-06415-7)
Supplement: Supplementary file 1 — Supplementary Material 1 (DOCX 595 KB) [file 894_2025_6415_MOESM1_ESM.docx]

**SUPPLEMENTARY INFORMATION**

**Noncovalent dyads of lanthanide nitride cluster fullerenes Ln_3_N@C_80_ and bisphthalocyanines LnPc_2_: Insights from DFT calculations**

Lina M. Bolivar-Pineda,^a,^* Elena V. Basiuk^a^ and Vladimir A. Basiuk ^b,^*

^a^ *Instituto de Ciencias Aplicadas y Tecnología, Universidad Nacional Autónoma de México, Circuito Exterior C.U., Ciudad de México 04510, México*

^b^ *Instituto de Ciencias Nucleares, Universidad Nacional Autónoma de México, Circuito Exterior C.U., Ciudad de México 04510, México*

__________________________________________________

*Corresponding authors:

E-mail: [lina.bolivar@icat.unam.mx](mailto:lina.bolivar@icat.unam.mx), [basiuk@nucleares.unam.mx](mailto:basiuk@nucleares.unam.mx)

**Table S1** Ln-N bond lengths (in Å), Ln-N-Ln angles (in °), the pyramidalization angle (θ) of the Ln_3_N clusters (in °) encapsulated in the C_80_ fullerene, noncovalently interacting with lanthanide bisphthalocyanines, as well as the corresponding values for each isolated Ln_3_N component. Also included are the shortest distances between the components of [Ln_3_N@C_80_](mailto:LaPc2+La3N@C80) + LnPc_2_ dyads, such as $N_{{LnPc}_{2}}$···$C_{C_{80}}$ (Å), $C_{{LnPc}_{2}}$···$C_{C_{80}}$ (Å) and ${Ln}_{{LnPc}_{2}}$···$C_{C_{80}}$ (Å)

| **System** | [**Ln_3_N@C_80_**](mailto:LuPc2+Lu3N@C80) | | | **Closest contacts** | | |
| --- | --- | --- | --- | --- | --- | --- |
|  | **Ln-N (Å)** | **Ln-N-Ln (°)** | **θ (°)** | $\mathbf{N}_{\mathbf{LnPc}_{\mathbf{2}}}$**···**$\mathbf{C}_{\mathbf{C}_{\mathbf{80}}}$ **(Å)** | $\mathbf{C}_{\mathbf{LnPc}_{\mathbf{2}}}$**···**$\mathbf{C}_{\mathbf{C}_{\mathbf{80}}}$ **(Å)** | $\mathbf{Ln}_{\mathbf{LnPc}_{\mathbf{2}}}$**···**$\mathbf{C}_{\mathbf{C}_{\mathbf{80}}}$ **(Å)** |
| [La_3_N@C_80_](mailto:La3N@C80)*^a^* | 2.260, 2.247, 2.265 | 94.6, 95.8, 96.0 | 31.3 |  |  |  |
| [Ce_3_N@C_80_](mailto:Ce3N@C80)*^a^* | 2.179, 2.223, 2.242 | 90.6, 93.5, 95.2 | 33.0 |  |  |  |
| [Gd_3_N@C_80_](mailto:Gd3N@C80)*^a^* | 2.064, 2.065, 2.066 | 118.2, 119.1, 119.5 | 5.9 |  |  |  |
| [Lu_3_N@C_80_](mailto:Lu3N@C80)*^a^* | 2.047, 2.049, 2.050 | 118.8, 119.8, 120.0 | 3.9 |  |  |  |
| [La_3_N@C_80_](mailto:LaPc2+La3N@C80) + LaPc_2_ | 2.237, 2.257, 2.265 | 96.4, 97.5, 98.6 | 29.7 | 2.975 | 2.889 | 4.271 |
| [Ce_3_N@C_80_](mailto:CePc2+Ce3N@C80) + CePc_2_ | 2.147, 2.136, 2.165 | 103.7, 104.1, 106.5 | 23.8 | 2.979 | 2.904 | 4.190 |
| [Gd_3_N@C_80_](mailto:GdPc2+Gd3N@C80) + GdPc_2_ | 2.094, 2.110, 2.110 | 110.3, 114.0, 114.1 | 15.9 | 2.884 | 2.967 | 4.250 |
| [Lu_3_N@C_80_](mailto:LuPc2+Lu3N@C80) + LuPc_2_ | 2.048, 2.062, 2.063 | 115.5, 121.4, 121.6 | 4.1 | 2.948 | 2.946 | 4.055 |

*^a^* Data reported in reference [1]

**Table S2** Total energies (E_total_ in Ha), formation energies (ΔE in kcal/mol), HOMO, LUMO and HOMO-LUMO gap energies (in eV) for Ln_3_N@C_80_ + LnPc_2_ dyads and their isolated components Ln_3_N@C_80_ and LnPc_2_

| **System** | ***E*_total_ (Ha)** | **Δ*E* (kcal/mol)** | **HOMO (eV)** | **LUMO (eV)** | ***E*_gap_ (eV)** |
| --- | --- | --- | --- | --- | --- |
| [La_3_N@C_80_](mailto:La3N@C80)*^a^* | -3272.137281 |  | -6.012 | -4.640 | 1.371 |
| [Ce_3_N@C_80_](mailto:Ce3N@C80)*^a^* | -3301.948865 |  | -5.818 | -4.956 | 0.862 |
| [Gd_3_N@C_80_](mailto:Gd3N@C80)*^a^* | -3690.918915 |  | -6.046 | -4.589 | 1.457 |
| [Lu_3_N@C_80_](mailto:Lu3N@C80)*^a^* | -5051.664737 |  | -6.034 | -4.506 | 1.528 |
| LaPc_2_*^b^* | -3388.129906 |  | -4.810 | -4.679 | 0.131 |
| CePc_2_*^b^* | -3398.064808 |  | -4.697 | -4.501 | 0.196 |
| Gdc_2_*^b^* | -3527.718856 |  | -4.760 | -4.630 | 0.130 |
| [LuPc_2_](mailto:LuPc2+Lu3N@C80)*^b^* | -3981.333451 |  | -4.654 | -4.517 | 0.137 |
| [La_3_N@C_80_](mailto:LaPc2+La3N@C80) + LaPc_2_ | -6660.337395 | -44.06 | -5.011 | -4.880 | 0.131 |
| [Ce_3_N@C_80_](mailto:CePc2+Ce3N@C80) + CePc_2_ | -6700.075195 | -38.61 | -4.850 | -4.661 | 0.189 |
| [Gd_3_N@C_80_](mailto:GdPc2+Gd3N@C80) + GdPc_2_ | -7218.710541 | -45.66 | -4.922 | -4.791 | 0.131 |
| [Lu_3_N@C_80_](mailto:LuPc2+Lu3N@C80) + LuPc_2_ | -9033.068986 | -44.43 | -4.869 | -4.735 | 0.135 |

*^a^* Data reported in reference [1]

*^b^* Data reported in reference [2]

| **System** | [**Ln_3_N@C_80_**](mailto:LuPc2+Lu3N@C80) | | | | **LnPc_2_** | |
| --- | --- | --- | --- | --- | --- | --- |
|  | **Ln charge (*e*)** | **N charge (*e*)** | **Ln spin (*e*)** | **N spin (*e*)** | **Ln charge (*e*)** | **Ln spin (*e*)** |
| [La_3_N@C_80_](mailto:La3N@C80)*^a^* | 0.447, 0.467, 0.486 | -0.925 | 0, 0, 0 (0)*^c^* | 0 |  |  |
| [Ce_3_N@C_80_](mailto:Ce3N@C80)*^a^* | 0.397, 0.415, 0.439 | -0.809 | -0.948, 0.992, 1.044 (1)*^c^* | -0.042 |  |  |
| [Gd_3_N@C_80_](mailto:Gd3N@C80)*^a^* | 0.496, 0.512, 0.527 | -0.992 | 6.899, -6.934, -6.935 (7)*^c^* | 0.028 |  |  |
| [Lu_3_N@C_80_](mailto:Lu3N@C80)*^a^* | 0.557, 0.584, 0.595 | -1.074 | 0, 0, 0 (0)*^c^* | 0 |  |  |
| LaPc_2_*^b^* |  |  |  |  | 1.827 | 0 |
| CePc_2_*^b^* |  |  |  |  | 1.719 | 0.001 |
| Gdc_2_*^b^* |  |  |  |  | 1.452 | -7.007 |
| [LuPc_2_](mailto:LuPc2+Lu3N@C80)*^b^* |  |  | 0 |  | 1.400 | 0.002 |
| [La_3_N@C_80_](mailto:LaPc2+La3N@C80) + LaPc_2_ | 0.484, 0.512, 0.721 | -0.946 | 0, 0, 0 | 0 | 1.903 | 0 |
| [Ce_3_N@C_80_](mailto:CePc2+Ce3N@C80) + CePc_2_ | 0.421, 0.473, 0.638 | -0.897 | -1.019, -1.019, -1.036 | 0.093 | 1.779 | -0.001 |
| [Gd_3_N@C_80_](mailto:GdPc2+Gd3N@C80) + GdPc_2_ | 0.502, 0.557, 0.568 | -0.955 | -6.906, 6.944, 6.988 | -0.028 | 1.474 | -7.011 |
| [Lu_3_N@C_80_](mailto:LuPc2+Lu3N@C80) + LuPc_2_ | 0.602, 0.636, 0.688 | -1.046 | 0, 0, 0 | 0 | 1.428 | 0.002 |

**Table S3** Charge and spin (in *e*) for the Ln and N atoms in the Ln_3_N cluster for Ln₃N@C_80_ + LnPc_2_ dyads, compared to the corresponding values in the isolated Ln_3_N@C_80,_ as well as charge and spin for Ln atom in LnPc_2_

*^a^* Absolute values reported in reference [1]

*^b^* Data reported in reference [2]

*^c^* Spin of isolated Ln^3+^ ion in parenthesis.

**
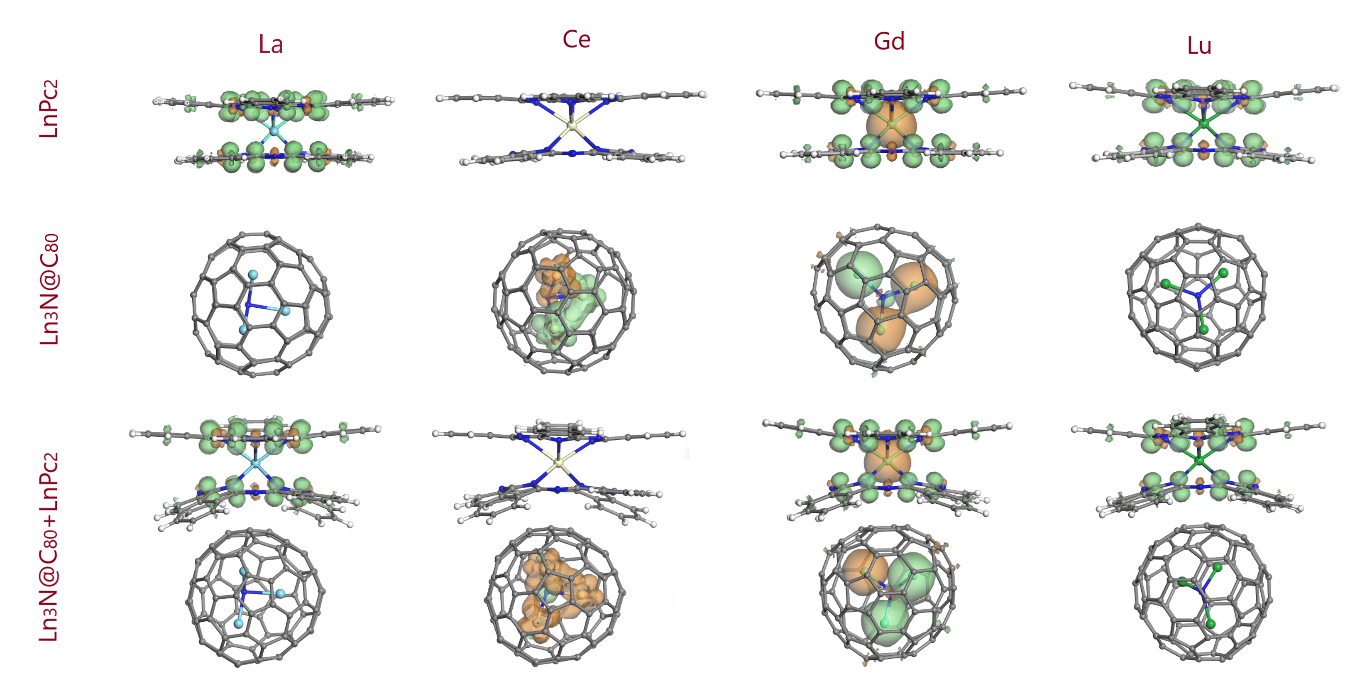
**

**Fig. S1** Comparison of spin density patterns (isosurfaces at 0.01 a. u.) for isolated LnPc_2_ and Ln_3_N@C_80_ molecules with those for their noncovalent dyads Ln_3_N@C_80_ + LnPc_2_. The green and orange lobes correspond to spin-up and spin-down electrons, respectively

**References**

1. Martínez-Flores C, Basiuk VA (2023) DFT Analysis of the Electronic and Structural Properties of Lanthanide Nitride Cluster Fullerenes Ln_3_N@C_80_. Inorganics 11:223. https://doi.org/10.3390/inorganics11050223

2. Martínez-Flores C, Bolívar-Pineda LM, Basiuk VA (2022) Lanthanide bisphthalocyanine single-molecule magnets: A DFT survey of their geometries and electronic properties from lanthanum to lutetium. Mater Chem Phys 287:126271. https://doi.org/10.1016/j.matchemphys.2022.126271
